# The effects of morning priming exercise on afternoon physical and cognitive performance in female field hockey players

**DOI:** 10.1371/journal.pone.0349645

**Published:** 2026-06-15

**Authors:** Jamie Knight, Mark Russell, Daniel Cunningham, Natalie Brown, Christian Cook, Mark Waldron, Laura Mason, Liam Kilduff

**Affiliations:** 1 Applied Sports Technology Exercise and Medicine Research Centre (A-STEM), Faculty of Science and Engineering, Swansea University, Swansea, United Kingdom; 2 Team Durham, Durham University, Durham, United Kingdom; 3 Faculty of Health, Wellness and Life Sciences, Leeds Trinity University, Leeds, United Kingdom; 4 Welsh Institute of Performance Science (WIPS), Swansea University, Swansea, United Kingdom; 5 Biomedical Discipline, School of Science and Technology, University of New England, Armidale, NSW, Australia; 6 Hamlyn Centre, Imperial College, London, United Kingdom; Ordu University, TÜRKIYE

## Abstract

**Objective:**

Hockey players concurrently experience physical and cognitive fatigue during competition, yet these are critical for successful performance. Prior research has shown cognitive and physical impairments after hockey matches. Morning resistance training may enhance afternoon neuromuscular and cognitive performance via diurnal changes in hormonal status. This study aimed to examine the effects of morning resistance exercise on afternoon physical and cognitive performance in field hockey players**.**

**Methods:**

On two separate occasions (randomised crossover design), 19 university female hockey players (19 ± 1 years) completed morning assessments of physical performance (countermovement jump, 40 m linear sprint) and cognitive function (rapid visual information processing, spatial working memory, paired associates of learning). Control (passive rest) or intervention (barbell back squat, 3 x 3 repetitions at 85% of one repetition maximum and barbell squat jump, 5 x 3 repetitions at 40% one repetition maximum) were implemented 5.5 h before afternoon assessments.

**Results:**

Afternoon peak power output and jump height improved following intervention and control (*P* < 0.05). Peak power output and jump height improvements were greater following intervention (7.46% and 13.52% respectively) relative to control (3.16% and 4.85% respectively). Cognitive and sprinting performance, and readiness to perform were unaffected by the intervention but did improve from morning to afternoon.

**Conclusion:**

Morning lower-body heavy and ballistic resistance exercise enhanced afternoon physical performance markers but did not affect cognitive performance in female hockey players.

## Introduction

Field hockey is a multifactorial, complex sport requiring high levels of technical, tactical and physical qualities [1]. Success is underpinned by players anticipating, adapting and performing successfully whilst potentially under extreme time pressure, and concurrently experiencing physical and cognitive fatigue [1,2]. High speed movements (³15.1 km × h^-1^) can contribute to approximately 19% (1070 m) of total distances covered by elite female players within a match [3]. These high-speed movements include decisive moments, such as forwards continuously seeking scoring opportunities by sprinting into space in the opposition half [1,4]. Similarly, midfielders serve as a crucial tactical link between defence and attack, both in and out of possession, and have been shown to cover more high-speed distances compared to all other positions [1]. Alongside these physical demands, midfielders face considerable cognitive challenges during match play [2]. Working memory (WM) plays an essential role in managing these demands, as midfielders must constantly update and manipulate information in dynamic game environments. For example, they are required to track the positions of multiple teammates and opponents and make rapid decisions regarding passing options under pressure [2]. Additionally, WM supports anticipation of opponents’ movements, switching attention between defensive and offensive responsibilities. Previous research has demonstrated impairments in WM following competitive matches [2]. Furthermore, mental fatigue has been found to reduce accuracy in high-level athletes [5,6]. This underscores the intense physical and cognitive demands placed on female field hockey players.

The acute enhancement of physical and potentially cognitive performance is possible on match-day through methods such as resistance training [7]. For the purposes of this study resistance training is defined by the NSCA as ‘to a specialised method of conditioning, which involves the progressive use of a wide range of resistive loads and a variety of training modalities designed to enhance health, fitness, and sports performance’ [8]. Specifically, morning resistance-training (RT) conducted five to six hours prior to subsequent exercise has displayed efficacious improvements in indices of afternoon neuromuscular performance [9–13]. Improvements in afternoon countermovement jump (CMJ) peak power (+2.7%) and jump height (2.5%) has occurred in male rugby and cricket players [9,13] when preceded by high intensity resistance training (> 85% one repetition maximum) within six hours. Further to this Nutt et al [13] demonstrated enhanced sprinting performance (1.2%) 5.5 h post morning RT (trap bar deadlifts, 6 x 4 repetitions up to 85% repetition maximum; RM). The mechanisms underpinning performance enhancements following morning RT are likely multifactorial, involving physiological, neural and psychological processes [14]. For example, acute increases in muscle-tendon stiffness following priming exercise may enhance rate of force development and power output by optimising fascicle-tendon interactions during high velocity actions, potentially mediated by early inflammatory responses [14]. Additionally, peripheral mechanisms, such as increased fibre sensitivity to calcium ions, have been proposed, though these remain to be fully investigated [11]. Likewise, the literature proposes diurnal changes in hormonal status may contribute to performance benefits following morning RT [11]. Testosterone follows a circadian rhythm, peaking in the morning and gradually declining over the day [11]. Research has shown performing morning resistance exercise can attenuate this decline, with elevated serum testosterone concentrations persisting for up to six hours and contributing to enhanced afternoon performance [9,10].

Subcomponents of cognition, such as perception, memory, attention, and executive function, all play a vital role in achieving successful skill execution and team sports performance [2,15]. In hockey, perception has been identified as a critical factor in a goalkeeper’s ability to successfully defend against penalty corner attempts [16,17]. These findings are also relevant to outfield players, who must quickly react to the ball’s position or the movements of an opponent. Working memory encompasses a player’s ability to retrieve information from both immediate experiences, such as a recent interaction on the field, and longer-term experiences, including tactical instructions provided before the match [18]. Hockey players must effectively scan the field, recognise and recall opposition attacking patterns, and respond efficiently to various cues. It has been reported that basketballers with greater WM have better tactical decision making and inhibition of irrelevant auditory cues [19]. Research has demonstrated an opportunity to acutely improve WM and attention following resistance exercise [20,21]. However, additional research is needed to examine the impact of acute WM enhancement through morning RT.

The effect of morning RT priming on cognitive performance remain uncertain [10], especially in female athletes [22]. Reductions in reaction time performance have been found 2 h post priming within elite female rugby players when compared to control [22]. Similarly in male rugby players, Russell et al [10] found no significant improvements in reaction performance following morning RT. Comparatively, Nutt et al [13] reported improved Stroop test performance (−3.83 s, 7.4%) compared to control following the completion of morning RT within professional male cricketers. Mechanistically, testosterone has been linked to cognitive performance in females [23]. Some evidence exists demonstrating testosterone concentrations can be elevated acutely through resistance exercise in women [24]. Mitigating the natural diurnal decline in testosterone may potentially safeguard performance levels later in the day [9,10].

It is also important to acknowledge the current lack of research exploring the neuromuscular effects of priming interventions in female athletes. While previous mixed-sex studies have reported improvement in physical performance, they have not conducted sex-specific analyses [25,26]. Subsequently, current priming recommendations are largely based on research conducted in male populations, highlighting the need for further studies to establish effective priming strategies for female athletes. The primary aim of this study was to investigate the acute effects of a morning RT session on afternoon physical and cognitive performance in female field hockey players. Specifically, the study sought to assess the impact of morning RT on neuromuscular performance, sprint performance, and cognitive function Based on previous research regarding the ergogenic effects of resistance-based priming, it was hypothesised that morning RT would elicit significant improvements in afternoon physical and cognitive performance compared to the control condition.

## Methods

### Subjects

Following institutional ethical approval (Swansea University Ethics Committee; 7344), 19 amateur female hockey players (age 19 ± 1 years, body mass 62.6 ± 5.2 kg, stature 1.55 ± 0.08 m) volunteered to participate in the current study following a recruitment period (from 08/8/2024–15/8/2024). An a priori ANOVA: Repeated-measures, within-factors power calculation was performed (G*Power version 3.1.9.6). A sample size of 15 was determined to be sufficient to detect a large effect size (Cohen’s f = 0.4) at 95% power (1 − β) and an alpha (α) of 0.05. This effect size was selected to ensure the study was powered to identify changes of high practical relevance to elite performance, consistent with robust ergogenic responses observed in acute resistance priming literature. All participants were healthy, injury-free and provided written informed consent prior to involvement.

Participants completed a menstrual cycle survey upon arrival at the testing facility. The survey was constructed in accordance with the bronze tier classification by Smith et al [27]. Participants were asked whether they were currently using hormonal contraceptives or undergoing a natural menstrual cycle, and if the latter, to identify the length of their cycle. Survey results were reviewed and of the 19 participants, one participant experienced irregular cycles and three were using hormonal contraceptives, specifically oral contraception and were therefore excluded from analysis. Of the remaining 15 participants experiencing regular cycles, they were categorised into either the follicular or luteal phase for each assessment day. Participants were assigned to one of the phases based on the number of days elapsed since the first day of their most recent menstrual bleeding [28]. The majority (79%) of the participants remained in the same phase across both assessment days, with only 21% of participants changing phases across the assessment days.

### Design

**Fig 1:**
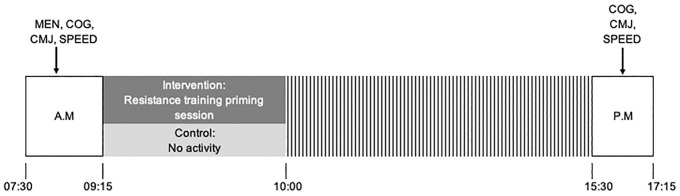
Overview of study protocol. MEN = menstrual cycle survey, COG = Paired associates of learning, spatial working memory, and rapid visual information processing assessments; CMJ = counter-movement jump; SPEED = 40 m linear sprint; = passive recovery; **A.**M = morning base measures; **P.**M = afternoon measures.

Following familiarisation, participants completed a control (CON) and intervention (INT) assessment day in a randomised, counterbalanced, cross-over design. Each assessment day was separated by six days with participants completing their habitual training consisting of four pitch sessions and three gym sessions with no matches throughout this period. To minimise potential carryover effects from this training volume, the weekly schedule was standardised across the duration of the study, ensuring that each participant entered each assessment day following an identical 48-hour recovery and training load profile. Participants were asked to arrive in a fasted state and to abstain from caffeine, creatine, alcohol, strenuous exercise and any cognitively demanding tasks in the preceding 24 hours of each assessment day. Trial day timings (Fig 1) remained consistent across both assessment days to avoid the influence of diurnal variation in performance [29]. Participants were familiarised with the testing battery seven days prior to the study commencing and had over 12 months RT and maximal sprint training experi‌‌ence.

### Assessments

#### Cognitive performance assessment.

The Cambridge Neuropsychological Test Automated Battery (Cambridge Cognition Ltd.) (CANTAB) assessments were undertaken on an iPad in a quiet room with minimal distractions. Test administration was fully automated with on-screen text instructions and voiceover guidance provided within each assessment. CANTAB assessments are widely used within various clinical populations and have demonstrated their validity and test-retest reliability (0.68–0.89) with traditional neurocognitive tests [30–34]. The assessments in the current study consisted of the Paired Associates Learning (PAL), the Rapid Visual Information Processing (RVP) and the Spatial Working Memory (SWM) assessment completed in this order at all assessment points.

The PAL assessment was used to assess visual memory, new learning, and episodic memory [35]. The PAL is an 8-min assessment where boxes are displayed on the screen and open one-by-one in a randomised order to reveal patterns hidden inside. The patterns are then displayed in the middle of the screen, one at a time. Participants were required to touch the box where the pattern was originally located. Patterns are re-presented to remind the participant of their locations if an error was made. The RVP assessment was used for the assessment of sustained attention [35]. The RVP is a 7-min assessment where single digits appear one at a time at a rate of 100 digits per min. Participants were required to detect a series of target sequences and touch a button when they identified the last digit of a target sequence. The SWM assessment was used to assess working memory and executive functioning [35]. A 4-min assessment where the test begins with coloured boxes being shown on the screen. The aim of the test was that by touching the boxes and using a process of elimination, the participant should find one ‘token’ in each of the boxes and use them to fill up an empty column on the right-hand side of the screen. The colour and position of the boxes used are changed from trial to trial. Table 1 summarises all cognitive assessment outcomes along with their corresponding definitions.

**Table 1:** Cambridge Cognition cognitive assessment outcomes and definitions.

Test outcome	Outcome abbreviation	Definition
Paired associate learning total errors adjusted	PALTEA28	No. of times the subject chose the incorrect box for a stimulus on assessment problems, plus an adjustment for the estimated no. of errors they would have made on any problems, attempts and recalls they did not reach
Paired associate learning, first attempt memory score	PALFAMS28	No. of times a subject chose the correct box on their first attempt when recalling the pattern locations.
Paired associate learning, mean errors to success	PALMETS28	The mean no. of attempts made by a subject needed for them, to successfully complete the stage.
Rapid visual information processing response sensitivity	RVPA’	A’ prime is the signal detection measure of a subject’s sensitivity to the target sequence, regardless of response tendency.
Rapid visual information processing median response latency	RVPML	The mean response latency of a correct response to a target sequence.
Spatial working memory strategy	SWMSX	The no. of times a subject begins a new search pattern from the same box they started with previously. Insight into search strategy used by the subject.
Spatial working memory total errors, 4-, 6-, and 8-item	SWMTE468	Total errors: The total no. of times a box is selected that is certain not to contain a token and therefore should not have been visited by the subject for the 4, 6 and 8 token trials.
Spatial working memory total errors, 12-item	SWMTE12	Total errors: The total no. of times a box is selected that is certain not to contain a token and therefore should not have been visited by the subject for the trials with only 12 tokens.
Spatial working memory between errors, 4-, 6-, and 8-item	SWMBE468	Between errors: The no. of times the subject incorrectly revisits a box which a token has previously been found. Calculated across all 4, 6 and 8 token trials.
Spatial working memory between errors, 12-item	SWMBE12	Between errors: The no. of times the subject incorrectly revisits a box which a token has previously been found. Calculated across trials with 12 tokens only.

### Physical performance assessment

Physical performance was assessed via a CMJ and 40 m linear sprint. All jumps were performed on a Kistler force plate (model 9260AA, Kistler, Germany) and followed the previously recommended protocol for CMJ assessment [36]. Each CMJ commenced in a static standing position with arms akimbo, from which participants performed a preparatory dip before explosively jumping to attain maximum height. Participants performed three repetitions on each occasion, with the greatest jump height and peak concentric power achieved being retained for analysis. The 40 m linear sprint assessment was conducted on an artificial grass pitch used for training, with participants wearing their normal footwear on all occasions. Sprints commenced 0.5 m behind the start line, where participants waited for the verbal start command. Time taken to complete the 40 m sprint, along with a 10 m split time was recorded using electronic timing gates (Brower Timing Systems, USA) with the fastest of three completed sprints being retained for analysis.

### Procedures

Upon arrival at the testing facility, participants initially completed baseline subjective ratings of readiness via a scale adapted from Nutt et al [13] and Mason et al [37]. Participants documented their perceived ‘fatigue, ‘aggression’, ‘soreness’, ‘mood’, and ‘motivation’. Each were rated on a five-point Likert scale ranging from ‘1’ (least) to ‘5’ (most ready), to provide a score for each sub-scale and an overall readiness score. High scores indicate greater readiness. Following this, the CANTAB assessments were completed by participants.

Participants then completed a standardised dynamic warm-up, consisting of stretching and a 5-min moderate cycle, followed by the CMJ assessment. A standardised sprint warm-up followed by a 40 m linear sprint assessment on an artificial outdoor grass pitch was then conducted. During the study, the mean temperature was 12.4 ± 2.02 ºC and mean wind speeds were 8.04 ± 3.73 m/s (the MET Office), with daily maximum wind speeds ranging from 5.4 to 10.68 m/s across assessment days. To ensure environmental consistency, a randomised crossover design was employed so that any fluctuations in wind speed were distributed across both intervention and control conditions. Following this, participants underwent the priming intervention (INT) or the passive recovery period (CON) dependent on trial allocation. A 5.5-h passive recovery period followed for both INT and CON prior to participants completing afternoon testing using the same subjective ratings of readiness, cognitive and physical assessments in the same order as morning testing. Due to the effects of nutrition on cognitive performance [38], during the recovery phase participants were asked to ingest their habitual game day pre-match meal (approximately 1 g × kg^-1^ carbohydrates) followed by an afternoon carbohydrate rich snack, which was replicated on both assessment days. While adherence to these nutritional instructions was not formally verified, participants were briefed on the importance of replication, and verbal confirmation of compliance was obtained prior to the commencement of the second assessment session.

For INT, participants completed lower-body resistance exercise following a standardised warm-up (~8 min) that required completion of dynamic stretching and progressive cycling (Table 2).

**Table 2:** Priming intervention completed by participants.

Exercise	Sets	Reps	Intensity (% of 1 RM^a^)	Rest (minutes)
Barbell Back Squat	1	3	50	1
	1	3	75	3
	3	3	85	3
Barbell Squat Jump	5	3	40	3

^a^RM = repetition maximum

### Statistical analysis

Statistical analyses were carried out using SPSS Statistics software (IBM Inc., USA, version 29) with significance set at *P* ≤ 0.05 and data reported as mean ± standard deviation (SD). The Shapiro-Wilk test for normality was performed along with Mauchly’s test for sphericity with the Greenhouse-Geisser correction applied if the assumption of sphericity was violated. Two-way repeated measures ANOVA were performed to test for interaction effects (trial x time; A.M and P.M). Where significant interaction effects were observed, trial was deemed to have influenced responses and simple main effect analyses were undertaken. Significant differences were investigated via post-hoc Bonferroni-adjusted pairwise comparisons and partial eta-squared (h^2^) values. Paired samples t-tests were completed to discover any differences between time points and trials where significant interaction effects were found. Hedge’s g_av_ effect sizes (ES) were calculated for post-hoc comparisons and were interpreted as trivial (0.00–0.19), small (0.20–0.49), moderate (0.50–0.79), or large (≥ 0.80) [39,40]. Sprint performance analysis was conducted on 18 subjects due to 1 subject withdrawing from the final speed assessment. The outcome measures were categorised into primary and secondary endpoints to account for the breadth of the dataset. The primary outcomes were physical performance metrics, specifically CMJ height and peak power output, and sprint performance (10 m, 30 m fly, and 40 m). The secondary exploratory outcomes included cognitive function parameters (PAL, RVP, and SWM subscales) and subjective athlete readiness scores. Given the exploratory nature of the secondary cognitive and readiness assessments, these results are intended to generate hypotheses for future, more targeted research.

## Results

### Countermovement jump performance

**Fig 2:**
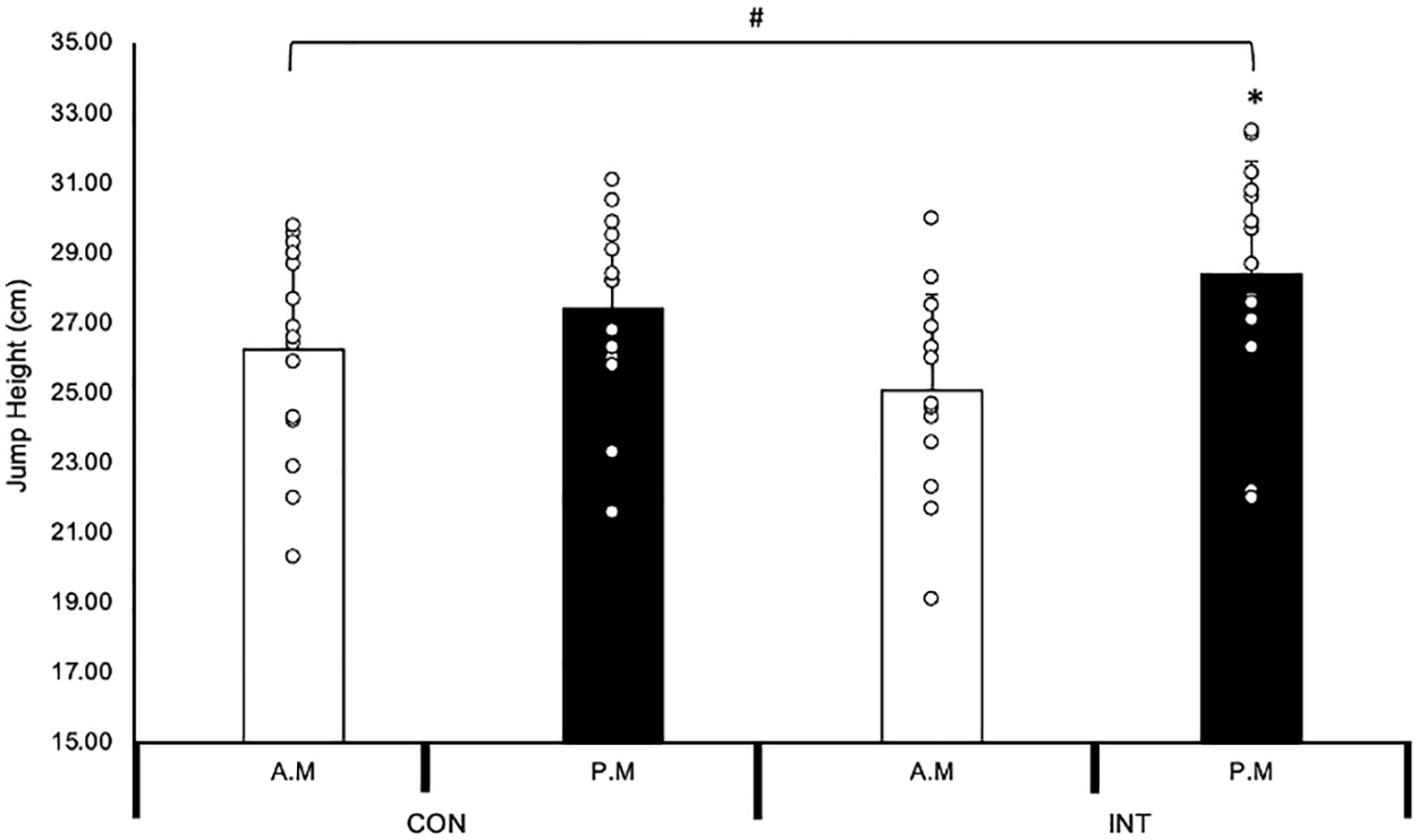
Mean ± SD countermovement jump height across conditions and time points.CON = Control, INT = Intervention. * = significant difference to A.M time point of trial condition. # = denotes the significant condition x time interaction (P < 0.05).

**Fig 3:**
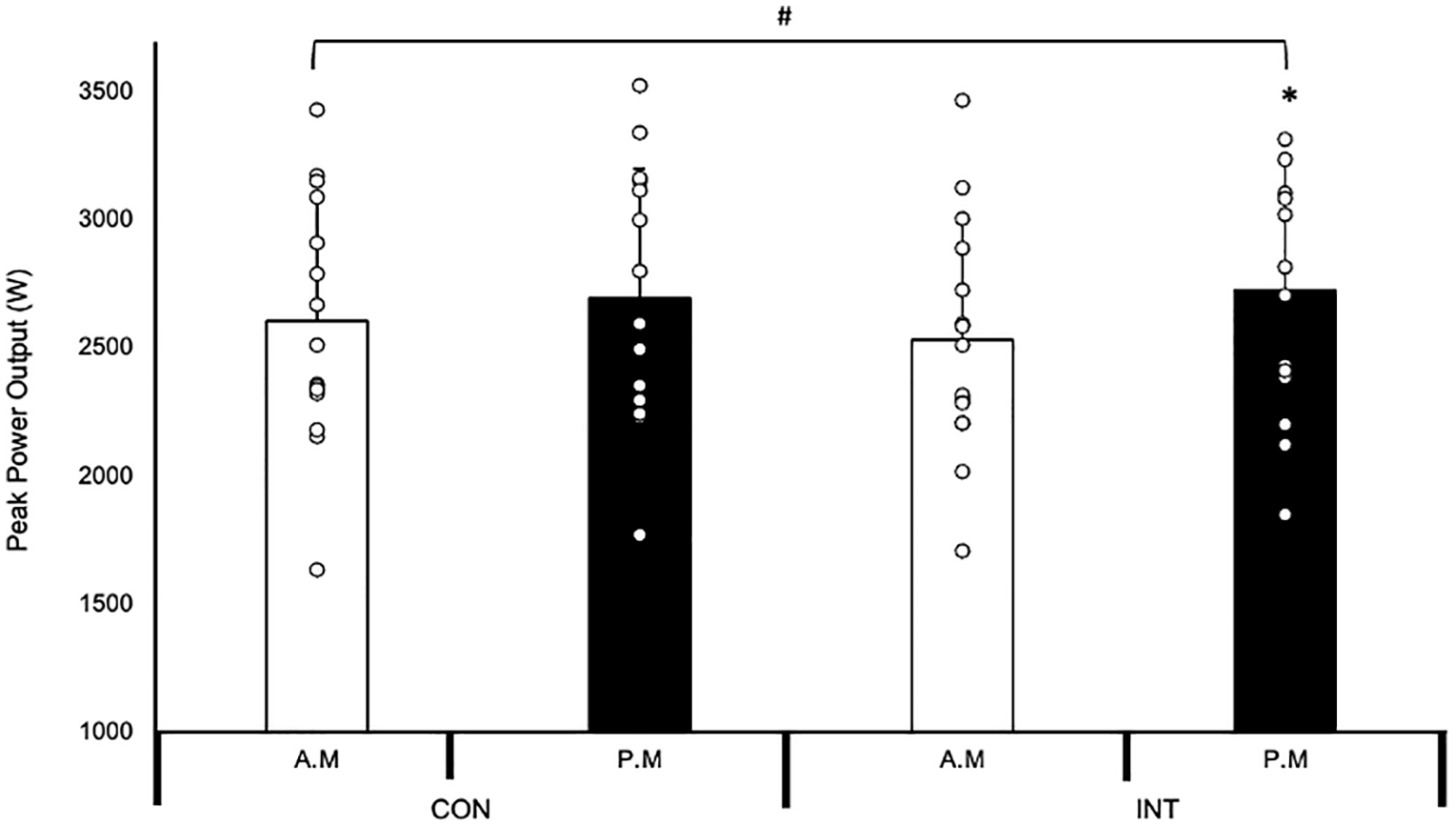
Mean ± SD countermovement peak power output across conditions and time points.CON = Control, INT = Intervention. * = significant difference to A.M time point of trial condition. # = denotes the significant condition x time interaction (P < 0.05).

Fig 2 and 3 presents the CMJ data as a function of condition. Significant interaction effects were found for countermovement jump height (JH) (*F* (1, 14) = 5.809, *P* = 0.03, h^2^ = 0.293) and peak power output (PPO) (*F* (1, 14) = 8.354, *P* = 0.012, h^2^ = 0.374). Delta analysis for A.M to P.M comparisons elicited superior mean changes in PPO and JH following INT compared to CON (Table 3). Time effects also existed for JH (*F* (1, 14) = 30.013, *P* < 0.001, h^2^ = 0.682) and PPO (*F* (1, 14) = 33.224, *P* < 0.001, h^2^ = 0.704), with mean P.M data exceeding A.M by 9.18 ± 8.74% and 5.31 ± 4.98% respectively.

**Table 3:** Delta analysis for countermovement jump performance.

Variable	Trial Condition	Δ	%	P-value	ES
Peak Power Output (W)	INT	196.71 ± 130.08	7.46 ± 4.94	0.006	−0.79, 95% CI [−1.34, −0.22]
	CON	80.96 ± 110.99	3.16 ± 4.13		
Jump Height (cm)	INT	3.35 ± 1.87	13.52 ± 7.62	0.003	−0.87, 95% CI [−1.44, −0.29]
	CON	1.16 ± 1.9	4.85 ± 7.75		

Data is presented as M ± SD. INT, intervention trial; CON, control trial; ES, effect size. Δ = Delta, represents the mean change in score from A.M to P.M. All analyses were conducted using a sample size *N* = 15. Differences between INT and CON deltas were analysed using paired-samples t-tests. Positive values indicate an improvement.

### Sprint performance

No significant interaction effects were found at any of the sprint distances (10 m, *F* (1, 13) = 1.667, *P* = 0.219, h^2^ = 0.114; 30 m, *F* (1, 13) = 0.618, *P* = 0.446, h^2^ = 0.045; 40 m, F (1, 13) = 1.291, *P* = 0.276, h^2^ = 0.090). Time effects were found at 10 m, 30 m and 40 m (Table 4). Mean speed times improved from AM to PM in 10 m (−0.06%), 30 m (−1.71%) and 40 m (−2.03%).

**Table 4:** Performance and readiness outcomes: Mean ± SD, delta analysis and time effect statistics (*P* values and partial η^2^).

Variable	A.M	P.M	Δ	P*-v*alue	η^2^
10 M (s)	1.93 ± 0.09	1.93 ± 0.09	−0.06%	<0.001	0.610
30 M (s)	4.43 ± 0.32	4.35 ± 0.29	−1.71%	<0.001	0.694
40 M (s)	6.43 ± 0.41	6.30 ± 0.38	−2.03%	<0.001	0.781
RVPA’	0.92 ± 0.06	0.94 ± 0.06	2.09%	<0.001	0.627
SWMSX	12.77 ± 5.31	11.23 ± 5.77	−12.01%	0.026	0.305
Fatigue	2.57 ± 0.9	3.57 ± 0.68	38.96%	<0.001	0.577
Aggression	2.23 ± 0.9	2.87 ± 0.9	28.36%	0.016	0.349
Motivation	2.77 ± 0.97	3.33 ± 0.92	20.48%	0.039	0.271
Soreness	3.53 ± 1.07	3.37 ± 0.81	−4.72%	0.313	0.072
Mood	3.07 ± 1.05	3.6 ± 0.81	17.39%	0.052	0.244

Data is presented as M ± SD. RVPA’, rapid visual information processing A’ prime; SWMSX, spatial working memory search strategy. η^2^, partial eta squared. Δ = Delta, represents the mean change in score from A.M to P.M conducted using a sample size *N* = 15.

### Cognitive performance

**Fig 4:**
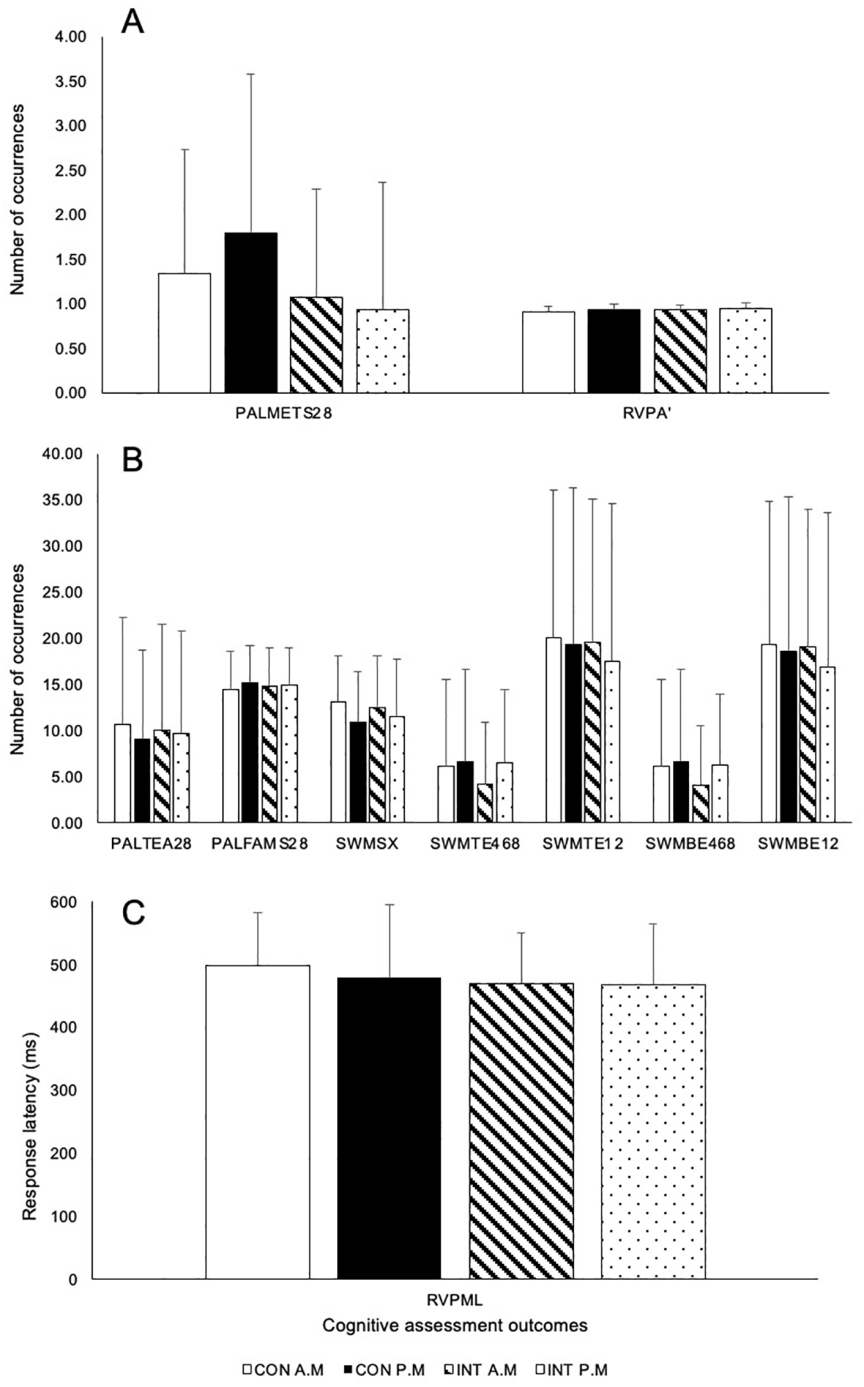
Mean ± SD cognitive assessment outcomes across conditions and time points. CON = Control, INT = Intervention. The results are categorised into three panels, panel A (PALMETS28 = Paired associate learning mean errors to success; RVPA’ = rapid visual information processing response sensitivity), panel B (PALTEA28 = Paired associate learning total errors adjusted; PALFAMS28 = Paired associate learning first attempt memory score; SWMSX = spatial working memory strategy; SWMTE468 = spatial working memory total errors 4-, 6-, and 8-item; SWMTE12 = spatial working memory total errors 12-item; SWMBE468 = spatial working memory between errors, 4-, 6-, and 8-item; SWMBE12 = spatial working memory between errors 12-item), and panel C (RVPML = rapid visual information processing median response latency).

Statistical analysis discovered no significant interaction effects for any indices of cognitive performance for PAL (PALTEA, *F* (1, 14) = 0.174, *P* = 0.683, h^2^ = 0.012; PALFAMS28; *F* (1, 14) = 0.187, *P* = 0.672, h^2^ = 0.013; PALMETS28; *F* (1, 14) = 0.566, *P* = 0.464, h^2^ = 0.039), RVP (RVPA’; *F* (1, 14) = 2.577, *P* = 0.131, h^2^ = 0.155; RVPML; *F* (1, 14) = 0.532, *P* = 0.478, h^2^ = 0.037) or SWM (SWMSX; *F* (1, 14) = 0.386, *P* = 0.545, h^2^ = 0.027; SWMTE468; *F* (1, 14) = 0.268, *P* = 0.613, h^2^ = 0.019; SWMTE12; *F* (1, 14) = 0.197, *P* = 0.664, h^2^ = 0.014; SWMBE468; *F* (1, 14) = 0.257, *P* = 0.620, h^2^ = 0.018; SWMBE12; *F* (1, 14) = 0.221, *P* = 0.646, h^2^ = 0.016) (Fig 4). Significant time effects were found for RVPA’ and SWMSX (Table 4). Mean values increased from AM to PM for RVPA’ (2.09%) and decreased for SWMSX (−12.01%).

### Readiness to perform

Two-way repeated measures ANOVAs discovered no significant interaction effect in overall subject readiness (*P* > 0.05). Time effects were found for the subscales; fatigue, aggression and motivation (Table 4). Mean values increased from AM to PM in fatigue (38.96%), aggression (28.36%), and motivation (20.48%).

## Discussion

This study investigated the effects of morning RT on afternoon physical and cognitive performance in female hockey players. Our findings showed significant improvements in peak power output and jump height in INT compared to CON, supporting previous research [13,41]. However, sprint and cognitive results showed no change following morning RT. The findings from the present study provide promising results on the effects of morning RT on afternoon physical performance within female athletes.

Relative to CON, lower body PPO (7.46 ± 4.94%) and JH (13.52 ± 7.62%) (Fig 2) improved 5.5 h after the completion of lower limb strength and ballistic exercise. These findings support the notion that morning RT can enhance afternoon performance in explosive activities in female team sport athletes [41]. Testosterone’s association with physical performance in female athletes has been previously documented [42]. However, as hormonal variables were not measured in the present study, no mechanistic conclusions can be drawn. While prior research suggests that transient hormonal responses to morning RT may occur [9–11], including attenuation of the typical diurnal decline in testosterone [9,10], the relevance of these responses to the current findings remains speculative. Therefore, although hormonal mechanisms may represent a potential explanation, this interpretation should be viewed with caution and cannot be confirmed within the context of the present data.

Previous research investigating the effect of priming in female cohorts has reported mixed findings [22,41,43]. Our findings agree with research conducted by Loturco et al [41], demonstrating lower body physical performance improvements following the prescription of morning RT. The researchers reported CMJ height improvements of 5.6% and 4% using 40% of 1RM and 80% 1RM, respectively, following a barbell jump squat priming session (6 x 6 repetitions). Comparatively, we report a mean improvement of 12.4 ± 7.8% in JH following morning RT (3 x 3 back squat, 5 x 3 squat jump). Although both studies adhere to the recommended low session volume of priming sessions [11], our findings demonstrate that performance improvements may be achievable with a lower session volume, which may be more favourable by athletes on competition day. Moreover, the present study utilised a complex training method, combining a heavy back squat followed by a ballistic squat jump in comparison to Loturco et al [41]. This method of training has been shown to be a successful way to chronically and acutely (< 10 min) improve force and power production [44]. However, the use of complex methods within the priming literature has not been well studied. Our results demonstrate that this method may be beneficial for female team sport athletes. However, future research should look to elucidate these findings within female athletes and other team sports.

Most priming research utilises vertical jump assessments to evaluate neuromuscular responses to priming interventions [11,12,45]. Limited studies have explored the impact of priming on afternoon sprint performance [9,10,40,46]. Given the sprinting demands in field hockey and team sports [3], assessing isolated sprint outcomes is a critical first step in understanding athlete readiness. While our study focused on single-effort sprint performance (10 m, 30 m, 40 m), it is recognized that athletes are frequently required to perform successive high-intensity efforts during match play. Our findings contribute to the limited body of research on sprinting performance, specifically within the female athlete population. While we did not observe an interaction effect for single sprint efforts, future research should explore whether priming interventions influence repeated sprint ability or the capacity to maintain speed over multiple efforts, which is equally vital for field-based athletes

The current literature lacks clarity on the effects of morning RT on cognitive performance [10,13,22], a notion supported by the findings of the current study. Improvements in reaction time (~7.4%) have been found in male cricketers following morning RT [13]. Contrary to this, previous research within elite female rugby players discovered no changes in reaction time following morning RT [22]. In the present study, no changes in reaction time were found, supporting the findings of Mason et al [22]. Additionally, no differences occurred for sustained attention and working memory, extending previous research to other required cognitive qualities in field hockey [2]. Mechanistically, the acute exercise-induced changes in cognitive performance are yet to be fully understood [47]. Metabolic and neurochemical hypotheses have been suggested to describe the exercise cognition interaction [48,49]. Evidence suggests that the influence of resistance exercise on cognitive function may follow an inverted-U relationship, where moderate intensities yield superior effects compared to low or high intensities [47,50–52]. Speculatively, the high-intensity prescription used in the present study (85% 1RM) may have been suboptimal for cognitive enhancement, despite being effective for physical priming. While the present study was not designed to test the inverted-U hypothesis across varying loads, our results align with suggestions that the intensities required to enhance cognition may differ from those needed for physical performance [22]. Interestingly, these findings contrast with those of Nutt et al. [13], where an identical 85% 1RM protocol elicited ergogenic cognitive effects in a different cohort. This discrepancy highlights that the optimal intensity for cognitive performance remains unclear and may be sex-dependent, though this remains an area for future systematic investigation.

As this study was not intended to examine menstrual cycle effects, and only 21% of participants changed cycle phase between assessment days, menstrual cycle phase was not statistically controlled for as a covariate in the analysis. Consequently, the potential influence of hormonal fluctuations on the observed performance outcomes was not evaluated. A meta-analysis by Jang et al [53] found no significant variations in cognitive performance throughout the menstrual cycle, despite physiological changes. Similarly, prior research has shown jumping and sprinting performance in trained women remains consistent, regardless of their menstrual cycle phase [54]. However, the area remains debated and dependent on more data [55].

Recognising the limitations of this study, incorporating hormonal and temperature analysis would have provided a deeper understanding of the potential mechanisms influenced by morning RT in female athletes. Moreover, the priming volumes applied in this study were primarily derived from research in males. Further research is needed to determine whether these recommendations are equally as effective for female athletes. Additionally, the limited ecological validity of the cognitive assessment battery used in this study must be acknowledged. Furthermore, the large number of interaction tests performed across physical and cognitive variables increases the risk of Type I error; therefore, significant findings for CMJ variables should be interpreted as exploratory, as they may no maintain significance under more conservative multiple-comparison corrections. Future research should consider evaluating cognitive performance through more ecologically valid methods, such as skill execution and error counts within the athletes’ respective sport. Considering the ongoing research around menstrual cycle influence on female athletic performance, future studies with stricter control of menstrual phase and hormonal status are warranted to better elucidate the relationship between priming interventions, physiological responses, and performance outcomes.

### Practical application

A morning RT session featuring both heavy strength exercises (barbell back squats, three sets of three repetitions at 85% of one repetition maximum) and ballistic exercises (barbell squat jumps, five sets of three repetitions at 40% one repetition maximum) has the potential to enhance afternoon physical performance, notably lower limb peak power output and vertical jump height when commenced 5.5 h post priming stimulus. Practitioners may wish to prescribe a high intensity strength exercise and a ballistic exercise on the morning of competition to improve afternoon physical performance.

## Conclusions

The current results indicate that afternoon physical performance can be enhanced following a 5.5-hour time interval in female hockey players. Conversely, our results did not reveal any significant improvements in sprint or cognitive performance. Future research should explore the effect of differing resistance intensities on cogniti‌‌ve performance.

## Supporting information

S1 File Table A Linear sprint test results, Table B Countermovement jumps test results, Table C Cognitive assessment test results, Table D Readiness to Perform survey results, Table E: Participant-reported menstrual cycle length. (XLSX)
